# Genetic diversity, population structure, and taxonomic confirmation in annual medic (*Medicago* spp.) collections from Crimea, Ukraine

**DOI:** 10.3389/fpls.2024.1339298

**Published:** 2024-04-03

**Authors:** Dongyan Zhao, Manoj Sapkota, Meng Lin, Craig Beil, Moira Sheehan, Stephanie Greene, Brian M. Irish

**Affiliations:** ^1^ Breeding Insight, Cornell University, Ithaca, NY, United States; ^2^ Agricultural Genetic Resources Preservation Research Unit, United States Department of Agriculture (USDA), Agricultural Research Service (ARS), Prosser, WA, United States; ^3^ Plant Germplasm Introduction and Testing Research Unit, United States Department of Agriculture (USDA), Agricultural Research Service (ARS), Prosser, WA, United States

**Keywords:** diversity, DArTag genotyping, marker, germplasm, legume

## Abstract

Annual medic (*Medicago* spp.) germplasm was collected from the Crimean Peninsula of Ukraine in 2008 to fill gaps in geographic coverage in the United States department of Agriculture, Agricultural Research Service, National Plant Germplasm System (NPGS) temperate-adapted forage legume collection. A total of 102 accessions across 10 *Medicago* species were collected. To assess genetic diversity, population structure, and to confirm taxonomic identities, the collections were phenotypically and genetically characterized. Phenotyping included the use of 24 descriptor traits while genetic characterization was accomplished using a 3K Diversity Array Technologies (DArTag) panel developed for alfalfa (*Medicago sativa* L.). For both field and molecular characterizations, a reference set of 92 geographically diverse and species-representative accessions were obtained from the NPGS collection. Phenotypic descriptors showed consistency among replicated plants within accessions, some variation across accessions within species, and evident distinctions between species. Because the DArTag panel was developed for cultivated alfalfa, the transferability of markers to the species being evaluated was limited, resulting in an average of ~1,500 marker loci detected per species. From these loci, 448 markers were present in 95% of the samples. Principal component and phylogenetic analysis based on a larger set of 2,396 selected markers clustered accessions by species and predicted evolutionary relationships among species. Additionally, the markers aided in the taxonomic identity of a few accessions that were likely mislabeled. The genotyping results also showed that sampling individual plants for these mostly self-pollinating species is sufficient due to high reproducibility between single (n=3) and pooled (n=7) biological replicate leaf samples. The phenotyping and the 2,396 Single Nucleotide Polymorphism (SNP) marker set were useful in estimating population structure in the Crimean and reference accessions, highlighting novel and unique genetic diversity captured in the Crimean accessions. This research not only demonstrated the utility of the DArTag marker panel in evaluating the Crimean germplasm but also highlighted its broader application in assessing genetic resources within the *Medicago* genus. Furthermore, we anticipate that our findings will underscore the importance of leveraging genetic resources and advanced genotyping tools for sustainable crop improvement and biodiversity conservation in annual medic species.

## 1 Introduction

The genus *Medicago* L. includes agriculturally significant crops like alfalfa (*M. sativa* L.) and the model legume *M. truncatula* L. Other important perennial and annual species useful as forages and cover crops are also in the genus. Many of the species are valuable because they cover the ground effectively preventing erosion and weeds, can help build soil organic matter content, are often a nutrient rich source of fodder, and can fix atmospheric nitrogen because of the ability to form a symbiosis with root nodulating bacteria. Most of the 87 currently described taxonomic species in the genus are of Eurasian or Mediterranean origin with ranges extending into Europe and North Africa (Small, 2011). Furthermore, due to the weedy nature of some species and extensive human-mediated dissemination, especially in the case of alfalfa, many of these species can now be found across the globe.

Alfalfa or lucerne is the most widely grown and important perennial forage legume crop worldwide (Undersander et al., 2021). In the United States, it was the fourth most cultivated crop in 2021 with an estimated direct value of $11.6 billion (Putnam and Meccage, 2022) and ranked first among forage crops by planting area with a total of 14.9 million acres in 2022 (https://www.nass.usda.gov/). Alfalfa is a key nutritional component for dairy and beef production because it contains a high amount of crude protein, provides dietary fiber needed to maintain rumen health, and is an excellent source of vitamins and minerals. In addition, it is unparalleled as a component of sustainable agricultural systems because of its perennialism, ability to fix nitrogen, protect water quality, interrupt pest and pathogen cycles in annual crops, and improve soil carbon storage (Fernandez et al., 2019). Alfalfa is a widely adapted plant that can grow in a range of environments, but its performance and adaptability can be influenced by factors such as climate, soil type, location, and management (Undersander et al., 2021). Alfalfa is a tetraploid, insect-pollinated, outcrossing crop, so it is highly heterogeneous and heterozygous with inherent genetic diversity. Improving and diversifying alfalfa cultivars is challenging, particularly concerning the effective enhancement of quantitative traits like yield. There is need to improve breeding strategies that harness the crop’s existing diversity to enhance key attributes. Furthermore, the exploration and utilization of novel genetic resources, including those of wild relatives, and the assessment of genetic diversity are of utmost importance for the sustainable development of improved alfalfa. Traits have been introgressed into cultivated alfalfa from several wild relatives (Mizukami et al., 2006; Humphries et al., 2021). However, challenges remain in utilizing biotechnology and molecular marker techniques to improve breeding efforts for alfalfa and related annual medic crops.

Of the thirty-five annual medic mostly diploid species described, several including barrel medic (*M. truncatula*), black medic (*M. lupulina*), burr medic (*M. polymorpha*), snail medic (*M. scutellata*), and strand medic (*M. litoralis*) have been used as forage crops and cover crops (Zhu et al., 1996; Fisk et al., 2001; Muir et al., 2006). Many of these annuals are an essential component of native Mediterranean region flora and as a fodder source (Piano and Francis, 1992; Small, 2011). They also have been introduced and used in southern Australian pasture systems with breeding efforts focusing on germination, vigor, adaptation to those edaphic conditions, and forage quality (Crawford et al., 1989; Nichols et al., 2012). Ideal growing conditions for many of the annual medics include warm dry summers with cooler winter periods, with well-drained light soils with neutral to basic pH levels (Zhu et al., 1996; Muir et al., 2006). Many of these species persist by producing hard seeds that germinate the following growing season (i.e., self-reseeding) leading to regenerating or ‘perennial’ pastures.

Genetic diversity conserved in plant germplasm collections is crucial for crop improvement, serving as the foundation for selection and breeding programs (Govindaraj et al., 2015). Novel genetic resources, characterized by unique traits and allelic variations, have significant potential for developing improved cultivars with enhanced productivity, stress tolerance, and nutritional quality. These resources are acquired through the collection projects and from other germplasm collections. Genebanks preserve and make this germplasm representing wild relatives, landraces, and varieties available (Gabriel, 1992; Guzzon et al., 2022). Genetic diversity within collections plays a crucial role in long-term crop sustainability. It provides resilience against environmental changes, pests, and diseases by maintaining a genepool of potentially advantageous alleles that can be selected under diverse pressures. Additionally, diverse populations are less susceptible to genetic erosion and offer a buffer against the loss of genetic adaptability and vulnerability to emerging threats (Mundt, 2002; Bijlsma and Loeschcke, 2012; King and Lively, 2012).

In the United States, the National Plant Germplasm System (NPGS) of the Department of Agriculture (USDA), Agricultural Research Service (ARS), is responsible for conservation and facilitation of access to a wide range of cultivated and wild plant genetic resources that are essential for the continued sustainability and advancement of plant agriculture (Postman et al., 2006; Byrne et al., 2018). A significant temperate-adapted forage legume (TFL) collection is managed as part of the NPGS collections in Pullman, WA. The collection includes larger subsets of accessions for important crops like alfalfa (>4,000 unique accessions), cultivated clovers (*Trifolium* spp. - e.g., red, and white clover), birdsfoot trefoil (*Lotus corniculatus* L.) as well as many wild relatives (Irish and Greene, 2021). Within the *Medicago* genus, large subsets of accessions are represented by many perennial and annual species, many of which are wild relatives of alfalfa and/or are crops themselves.

Assessing genetic diversity, population structure, reducing redundancy, and correctly assigning taxonomic identities in plant germplasm collections are important approaches to implement when managing plant genetic resource collections. Historically, highly heritable phenotypic traits have been used to address this need, with descriptor traits developed for many crops species (https://alliancebioversityciat.org/publications-data “Descriptors”) including the annual medics (International Board for Plant Genetic Resources (IBPGR), 1991). However, modern molecular marker approaches have evolved significantly and exceed as a tool for addressing these needs. Molecular marker techniques have been used for decades to study population structure and genetic diversity in plant germplasm collections in addition to enhance breeding efforts and accelerate genetic gains for major staple food crops (Tanksley, 1983; Helentjaris et al., 1985; Feuerstein et al., 1990; and reviewed in Hasan et al., 2021).

The development and use of molecular markers in forage crops like alfalfa and related *Medicago* species has been much slower than in other important agricultural food crops (Barker and Warnke, 2001). Various molecular and genomic techniques have been applied in alfalfa and other *Medicago* research. Molecular markers, such as random amplified polymorphic DNA (RAPD), restriction fragment length polymorphism (RFLP), amplified Fragment length polymorphism (AFLP), and simple-sequence repeats (SSRs), have been utilized to assess genetic diversity within different *Medicago* germplasm collections (Brummer et al., 1991; Kidwell et al., 1994; Musial et al., 2002; Maureira et al., 2004), construct linkage maps (Brummer et al., 1993; Brouwer and Osborn, 1999; Kaló et al., 2000), and conduct association studies (Barcaccia et al., 2000; Brouwer et al., 2000; Tavoletti et al., 2000). Nevertheless, these methods are labor-intensive and have become somewhat outdated. The emergence of next-generation sequencing technologies has introduced novel molecular approaches, including whole-genome sequencing and genotyping-by-sequencing (GBS), which facilitates the identification of genome-wide variants like single nucleotide polymorphisms (SNPs) and insertions and deletions (InDels) less than 20 bp. However, the computational demands are notably high, particularly for polyploid crops such as alfalfa. Considering these challenges, it is crucial to develop genomic tools tailored to the specific biological and logistical complexities of these crops. This tailored approach will have the potential to conveniently streamline the management of genetic resources and enhance breeding outcomes. One recently developed genotyping platform is DArTag (Diversity Array Technologies - DArT), which employs an amplicon-based targeted genotyping approach (Blyton et al., 2023; https://www.diversityarrays.com/services/targeted-genotying/). Oligos are designed to anneal to the target known genetic variants (SNPs and InDels) to produce sequencing products of 54 bp (legacy technology) or 81 bp (current technology) in length. The reads can be used to call SNPs, or in the case of complex genomes like alfalfa, used to identify microhaplotypes because sequencing reads can contain variants beyond the target SNP, which allows for the detection of more than two alleles at each of the 3,000 loci (Zhao et al., 2023). As the amplicons are very short, variants found within these reads are assumed to be in very strong linkage disequilibrium and therefore can be used for phasing genotyping calls and determining allele dosage. The practical maximum number of probes on a DArTag panel is ~3,000 loci, though, it’s worth noting that the optimal maximum may vary depending on the species and genome complexity, as well as the required read depths to accurately call genotypes (Andrzej Kilian, DArT, personal communication). One limitation of using targeted-amplicon sequencing platforms is the potential ascertainment bias (Heslot et al., 2013), which may restrict their applicability in diversity studies or when exploring species beyond those for which the marker panel was initially designed. Recognizing this issue, we view this study as a valuable test case to assess the panel’s effectiveness on other *Medicago* species related to alfalfa.

In this study, our goals were to comprehensively characterize the diversity of a subset of annual medic (*Medicago* spp.) accessions obtained from a plant germplasm collecting mission to the Crimean Peninsula of Ukraine. More specifically, we aimed to assess the phenotypic variation within and among different species, shedding light on the unique traits and characteristics exhibited by each accession. Secondly, we viewed this study as a valuable test case to assess the effectiveness of a 3K DArTag alfalfa marker panel for genotyping annual medics. Additionally, we described and characterized the genetic diversity and population structure of this collection, using the markers to unravel the relationships among the various annual medic species. By accomplishing these objectives, we aim to provide breeders access to valuable, previously unexplored genetic resources, along with a useful genotyping tool for studying population structure in annual medic species, thereby facilitating the enhancement of their breeding programs.

## 2 Materials and methods

### 2.1 Plant materials

**Figure 1:**
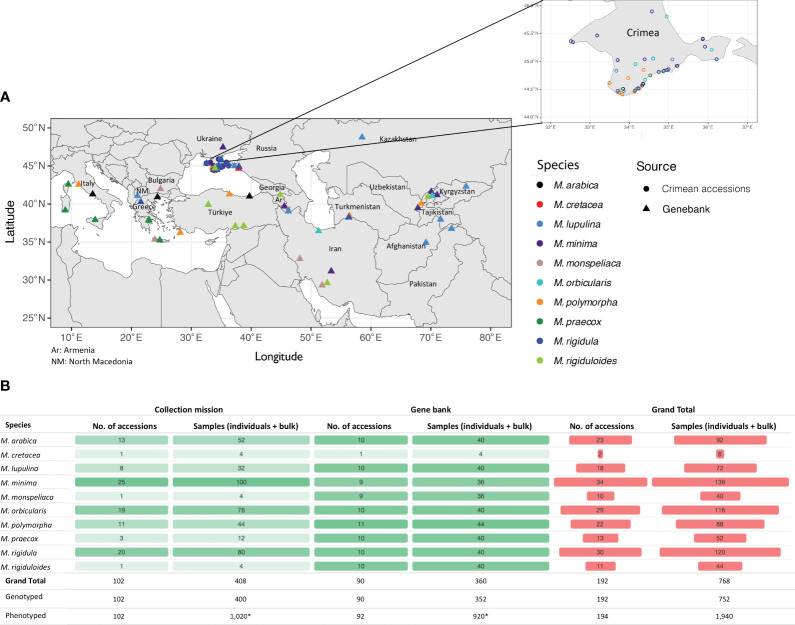
Summary information for *Medicago* spp. germplasm accessions used in the study. **(A)** Map showing the collection sites for most of the accessions used in the study. The inset shows information only for the accessions collected from Crimea, Ukraine. **(B)** Counts for accessions and number of samples genotyped from Crimea, Ukraine (Collection Mission) and the NPGS reference subset (Genebank). Each accession had three single-plant samples (biological replicates, n=3) and one bulked (n=7) sample. *10 plants for each accession were phenotyped in the field.

A total of 102 accessions across 10 *Medicago* species were collected along with their corresponding passport data from the Crimean Peninsula of Ukraine in 2008 (**Figure 1A** and **Supplementary Table 1**). The objective of the collecting trip was to fill gaps in the northern geographic range of several annual medic species, in particular, *M. truncatula*, which is used as an important genomic model species. The trip was a joint effort between the USDA NPGS, St. Petersburg State University, Russia, and the Ukrainian Genebank. A reference set of 92 accessions originating from geographically diverse regions of the world belonging to corresponding species available in the NPGS were also included in the study (**Figure 1A** and **Supplementary Table 1**).

Seeds for each accession were requested from the Pullman genebank, mechanically scarified, and 10 seeds for each accession were sown in bullet containers containing Sunshine Mix 3 soilless medium (Sun Gro, Agawam, MA) in early spring. Germinated seedlings were thinned to a single individual per container and were greenhouse-grown for approximately three months prior to field transplanting. Individual plants were established in non-replicated 10-plant field plots on the Washington State University (WSU), Irrigated Agriculture Research and Extensions Center (IAREC), Roza Research farm in May 2019. Annual medic descriptor data was collected in the field and laboratory for 24 traits (**Supplementary Table 2**). That descriptors included phenological and phenotypic information on vegetative and reproductive stages following published protocols for annual medics (International Board for Plant Genetic Resources (IBPGR), 1991). Descriptor data was collected by assigning visual ratings and by using measuring equipment such as rules, calipers, and laboratory balances. A mean quantitative or categorical trait was assigned across the representative 10-plant sample for each accession.

### 2.2 Tissue collection and genotyping

Prior to field establishment, young leaf tissues were collected from greenhouse seedlings, lyophilized, and stored at -18°C for further processing. A total of ten individual plants were sampled from each accession for DNA extraction. The leaves/leaflets were collected into labeled 1.5 ml microcentrifuge tubes and came from three individual plants (single-plant sample; n=3) and from an additional seven individual plants where leaves/leaflets were pooled (bulked sample; n=7). Appropriate starting mass of the lyophilized leaf tissues were transferred to 96-well plates for DNA extraction, which were performed inhouse using a DNeasy 96 Plant Kit (Qiagen, Valencia, CA) following the manufacturer’s instructions. Samples were transferred to DArT-required Eppendorf twin.tec^®^ microbiology Polymerase Chain Reaction (PCR) Plate 96 (Eppendorf, Enfield, CT). The DNA samples were then shipped to Diversity Array Technologies (DArT; www.diversityarrays.com) for genotyping.

Genotyping was performed using the recently developed alfalfa 3K DArTag panel (DArT product code: AlfaDArTagBICU; Zhao et al., 2023). This resulted in four genotyped samples (three single-plant samples and one bulked sample) for most accessions (**Figure 1B**). Altogether 768 samples across 192 accessions were genotyped using the DArTag panel. One *M. minima* and one *M. monspeliaca* accession from the reference set from NPGS were phenotyped but not genotyped. Briefly, the DArTag marker technology uses a targeted amplicon sequencing approach. Like other amplicon-based genotyping technologies, a short stretch of sequence (54-81 bp) around the target SNPs are captured, which allows for discovery of other off-target SNPs in addition to the reference and alternative microhaplotypes by assay design. Throughout the study, the genomic regions harboring the 3K SNPs were referred to as marker loci while the amplicons containing different combinations of the target and off-target SNPs from a marker locus were referred as microhaplotypes, which are provided in the DArTag report named missing allele discovery counts (MADC). For each marker locus, there could be varying numbers of microhaplotypes depending on the genetic diversity of the genotyped samples. It is worth noting that there may be some marker loci with paralogous sequences captured. However, these are usually with low read depths. Determining and eliminating paralogous microhaplotypes was out of scope for this study, therefore we expect some paralogous sequences to have been included in our final dataset.

### 2.3 Genotype processing

While processing the genotype in each of the sequenced samples, a read depth of one was considered a sequencing error for a single microhaplotype and was set to zero. To compare genetic diversity captured in single-plant samples with bulked samples, the total number of microhaplotypes were counted for each sample. For each of the accessions that had three single-plant samples and one bulked sample (n=181 accessions), the median total number of microhaplotypes among the three single-plant samples was used to represent their genetic diversity and was compared with the bulked sample for that accession. To investigate the overall trend of microhaplotype differences between single-plant and bulked samples, all accessions were ordered based on their bulked sample microhaplotype number. A locally weighted scatterplot smoothing (LOWESS) regression (Cleveland, 1979) was performed using microhaplotype numbers from single-plant samples in R. The same methodology was applied to compare the combined single-plant sample (by pooling sequences of all three single-plant samples) with the bulked sample in each accession. Across the entire panel and within each of the 10 species, the average number of microhaplotypes per marker was calculated for all single-plant samples and all bulked samples separately. These microhaplotype counts were further corrected by the total number of microhaplotypes of each marker in the entire population to scale values between 0 and 1.

**Figure 2:**
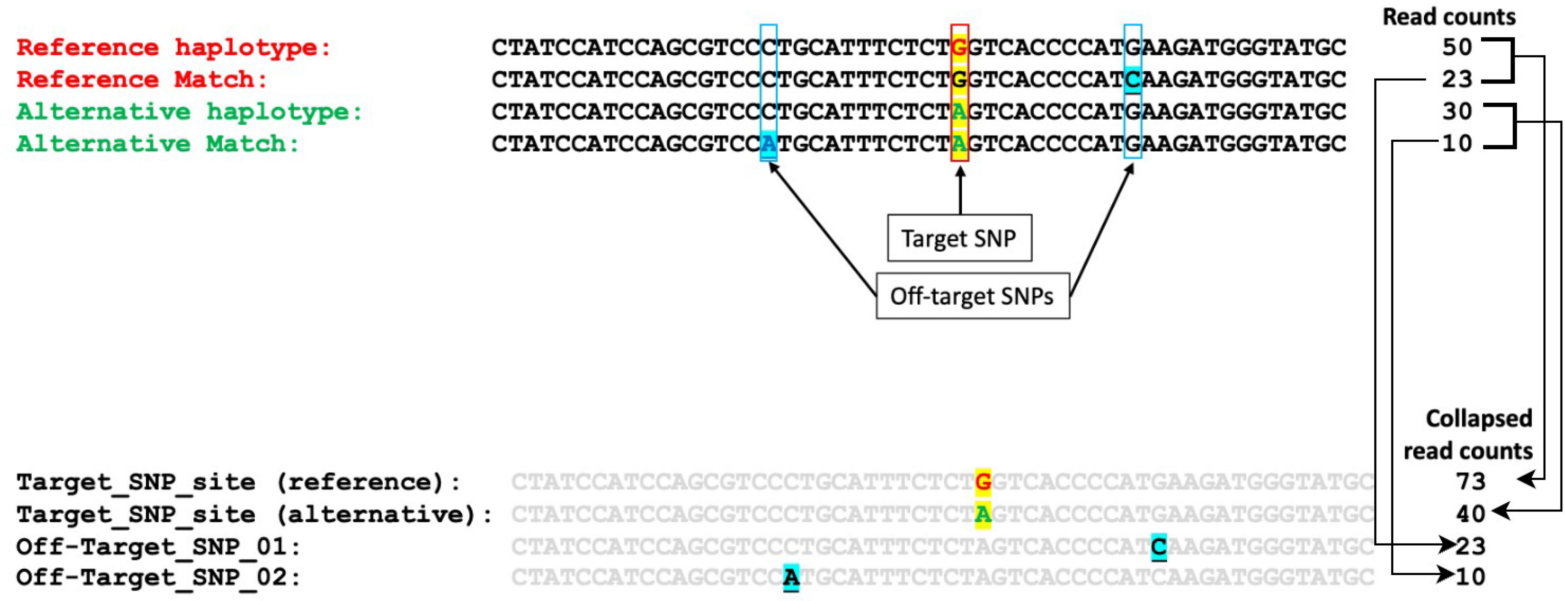
Illustration of the target and off-target SNPs extracted from microhaplotypes based on pairwise alignment with reference microhaplotypes and extraction of collapsed read counts from the individual read counts.

Marker loci with data (total read count > 10) in >5% of the samples were retained for genotype calling. Subsequent analyses were conducted using all SNPs, including both target and off-target SNPs, extracted from the microhaplotypes based on pairwise alignments with the reference microhaplotypes. SNP data were converted to variant call format (VCF) with the “FORMAT” field containing DP (total read depth), RA (reference allele read depth), and AD (read depth for each allele) (**Figure 2**). The polyRAD R package was used to estimate the ratio of individual heterzygosity to expected heterozygosity statistics (*H_ind_/H_E_*) at both the sample and SNP levels (Clark et al., 2019). The *H_ind_/H_E_* utilizes the likelihood that two sampled reads from a genotype will correspond to distinct alleles. A low *H_ind_/H_E_* value suggests the presence of alleles with low heterozygosity or sequencing errors, while a high *H_ind_/H_E_* value indicates that amplicons are likely derived from paralogous regions. In this study, only SNPs with a *H_ind_/H_E_* value between 0.1-1.0 were retained for subsequent analyses.

Furthermore, we acquired genotype information for 34 cultivated alfalfa accessions (**Supplementary Table 1**) utilized in the validation of the 3K DArTag panel from Zhao et al. (2023). These accessions underwent the same genotype processing procedures, primarily for the purpose of comparing missing marker loci and calculating genetic distances (as detailed in Section 2.5).

### 2.4 Descriptive statistics and statistical analysis

All the statistical analyses were performed in R (R Core Team, 2013), unless otherwise mentioned. Pairwise Pearson correlation coefficients were calculated using read counts per marker for replicates (three single-plant samples and a bulked sample) within each of the 192 accessions using the R function “cor()*”*. Prior to the analysis of variance (ANOVA) for missing marker loci, Levene’s test was performed to determine if the variances between each group were equal. The test was done using “leveneTest()” function in R package “car” (Fox et al., 2007). Welch’s ANOVA (using “welch_anova_test()” function in R package “rstatix” (Kassambara, 2019) was then performed to address the violation of assumption of equal variances. Post-hoc tests for Welch’s ANOVA were performed using Games-Howell test. Games-Howell test is a multiple mean comparison for Welch’s ANOVA. Briefly, it is used to compare all possible combinations of group differences when the assumption of homogeneity of variances is violated. This post-hoc test provides confidence intervals for the differences between group means and shows whether the differences are statistically significant (Games and Howell, 1976). All the figures were generated using ggplot2 package in R (Wickham, 2011). The upset plot was generated using “UpSetR” package in R (Conway et al., 2017). The plant germplasm accessions used in the study were georeferenced using R package “rnaturalearth” (South, 2017; https://CRAN.R-project.org/package=rnaturalearth) and “ggspatial” (Dunnington, 2023; https://CRAN.R-project.org/package=ggspatial). The package “rnaturalearth” uses the public domain map dataset “Natural Earth” (https://www.naturalearthdata.com/) to generate the base layer of the map.

### 2.5 Population structure and phylogeny

Population structure based on phenotype data was performed using principal component analysis (PCA) with “PCA()” function in “FactoMineR” package in R (Lê et al., 2008). PCA was performed using only the accession and phenotypes without any missing data in any trait recorded, resulting in 175 accessions and 18 traits for the PCA. The character variables recorded for phenotypic traits were converted to numeric by one-hot encoding using the “model.matrix()” function in the “stat” package in R (Team R Core, 2013). Furthermore, population structure based on genotype was assessed using PCA by implementing the “AddPCA()” function in the “polyRAD” package in R (Clark et al., 2019). PCA was performed using dosage-based calls using both the entire set of markers and a subset of 448 marker loci that were evenly distributed across the genome and found in >95% of the genotyped accessions. The clustering was based on the passport information of the germplasm. The phylogenetic tree was built using the Neighbor-Joining method (Saitou and Nei, 1987) and the Tamura-Nei genetic distance model (Tamura and Nei, 1993) with the entire genotype data and samples with the highest average read depth (altogether 192 samples) in Geneious Prime v2021.2.2 (Geneious Prime 2021; https://www.geneious.com/). Bootstrap support for the tree was obtained using 100 bootstrap replicates. The tree was then plotted using “ggtree” package in R (Yu et al., 2017). Weighted mean Fixation index (*F_ST_*) values were calculated for all species pair combinations using genotype calls in vcftools with “– weir-fst-pop” parameter (Danecek et al., 2011). Additionally, a phylogenetic tree based on genetic distance (Fixation index: *F_ST_*) was constructed using the R package “ape” (Paradis et al., 2004) and then visualized using the “ggtree” package (Yu et al., 2017).

## 3 Results

### 3.1 Phenotyping and population structure

**Figure 3:**
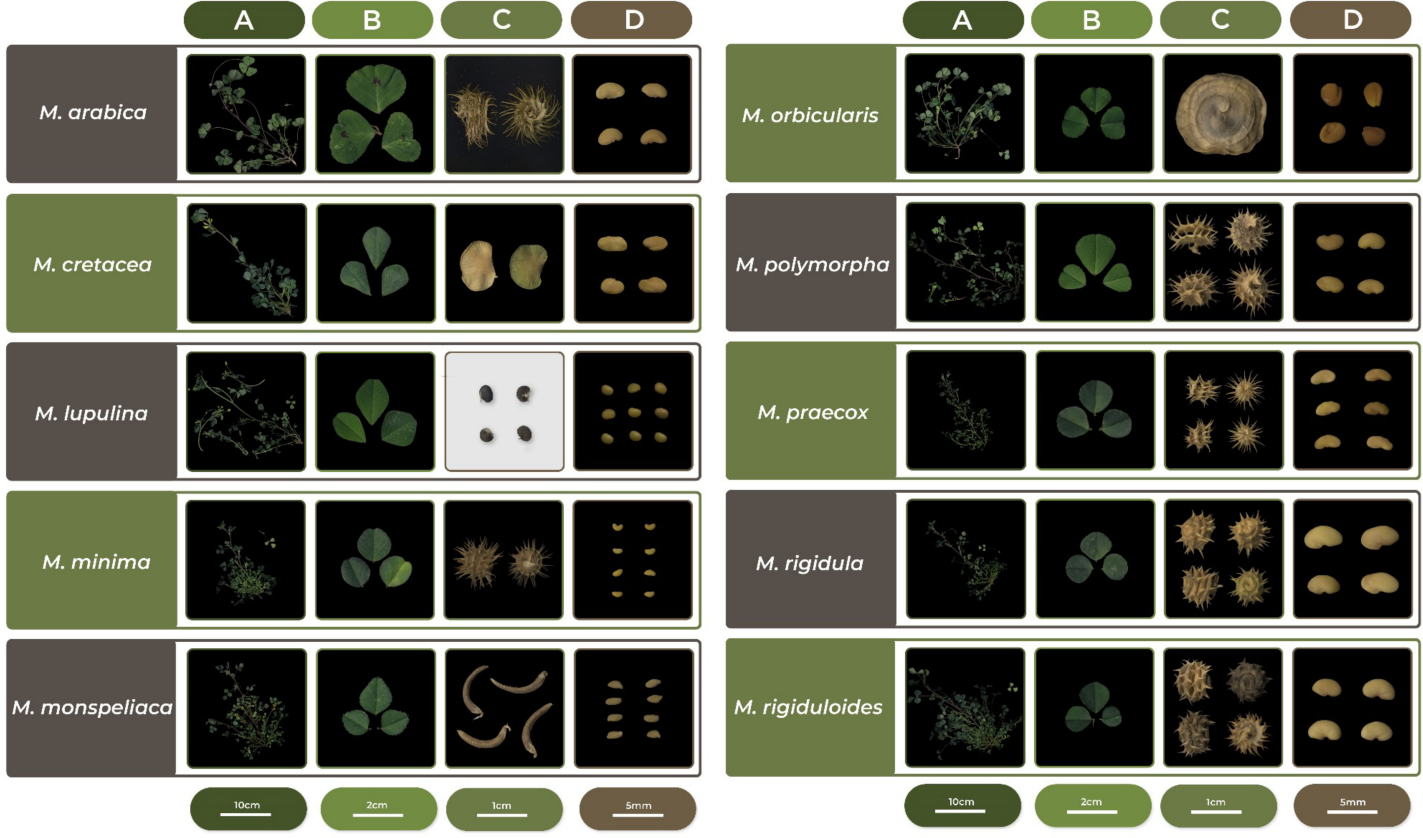
Representative phenotypic characteristics for the ten annual medic (*Medicago* spp.) species evaluated. **(A)** shoot architecture; **(B)** leaflets; **(C)** fruit/pods, and **(D)** seeds.

**Figure 4:**
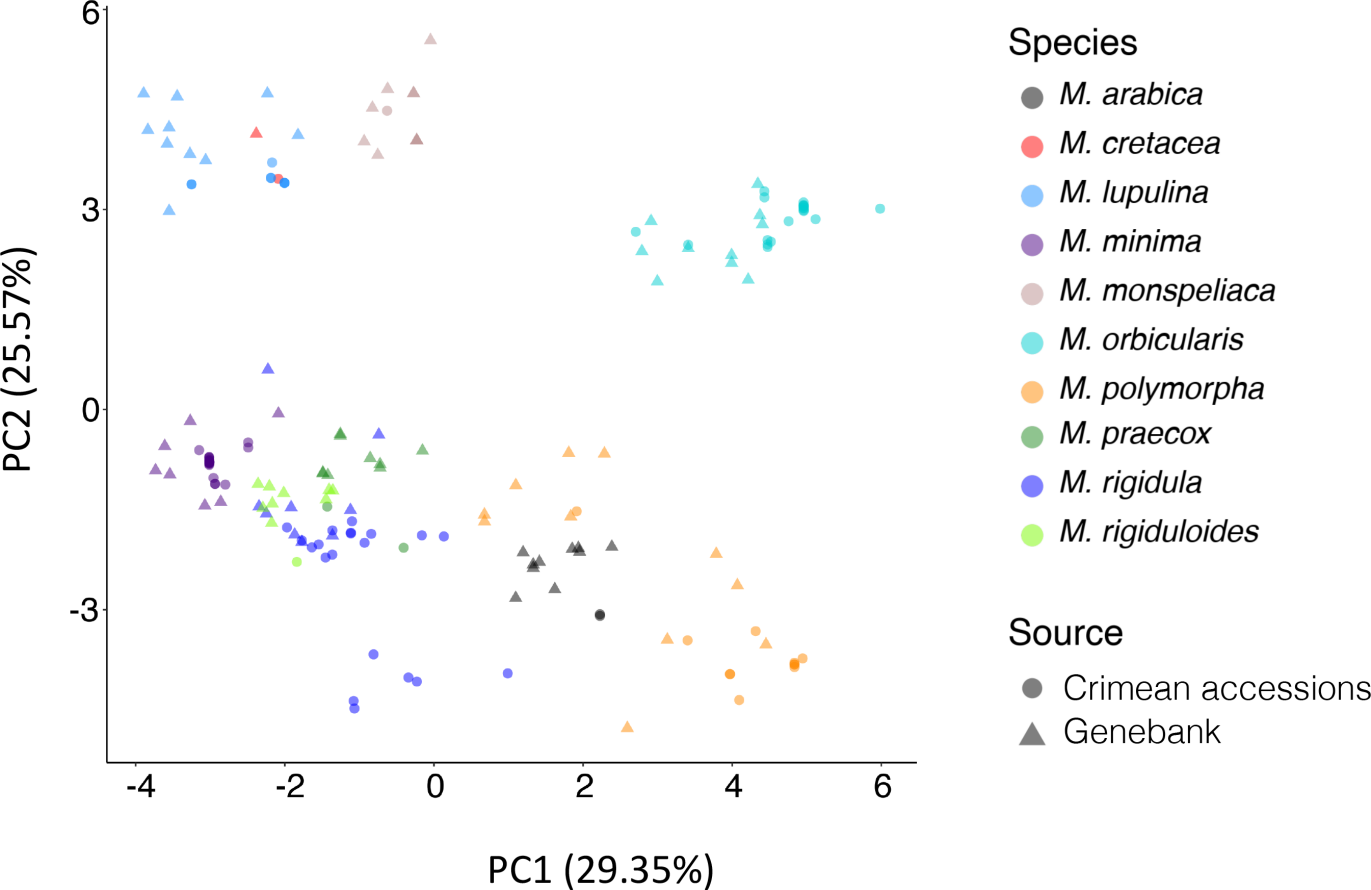
Principal component analysis based on 18 phenotypic descriptor traits of 175 accessions.

The corresponding 10 field-established plants for all 194 Crimea and reference accessions were used to collect 25 phenological and highly heritable phenotypic descriptors following guidelines published for annual medics (International Board for Plant Genetic Resources (IBPGR), 1991). Descriptors included flowering and harvest dates, plant growth habit, leaf shape and markings, and pod (fruit), and seed characteristics and were recorded following instructions in guidelines by visual assessment or by using basic laboratory equipment such as calipers and balances (**Figure 3**; **Supplementary Table 3**). In a few situations, accessions did not flower or produce pods, but other traits were collected. Although slight variation might have been evident for plants within accessions, an average trait was collected for the entire accession. For example, there might have been some differences in the growth habit trait for some of the 10 plants within an accession. Some more upright than others, but on average a semi-erect descriptor trait value was assigned. In a few instances, and only within reference accessions, off-type plants were identified and were considered mistakes and excluded in the average for those traits. The off types in these situations were likely seed mixed in by mistake during the cleaning process of a species not corresponding to the accessions being evaluated. Notes were kept on occurrence of off types in accessions to try to remedy and/or warn future requestors. Also, differences in descriptor traits across accessions within species were evident with this variation being more prominent across reference accessions as they originated from a broader geographic area. Lastly, most descriptors for each of the ten species evaluated matched those in the published literature. An exception was accession W6 5374 which was received from the genebank labeled as a *M. polymorpha*, but likely was *M. rigidula* or *M. rigiduloides* based on comparisons to descriptors collected for those species. PCA based on the phenotypic data resulted in general species-wise clustering. Accessions associated with species such as *M. orbicularis*, *M. monspeliaca*, *M. lupulina*, and *M. minima* exhibited distinct clustering, indicating notable morphological variations among these species (**Figures 3**, **4**). Conversely, certain species like *M. rigidula*, *M. rigiduloides*, and *M. praecox* were found to cluster together, suggesting a high degree of phenotypic similarity (e.g., coiled pods with spines) among accessions even across different species (**Figures 3**, **4**). Overall, these results underscore the presence of significant phenotypic diversity among the studied accessions.

### 3.2 Genotyping and filtering

Next, we aimed to investigate the genetic diversity underlying the observed phenotypic diversity in the studied accessions. However, no molecular makers and genotyping platforms are currently available for research on annual medics; therefore, we tested the utility of an alfalfa 3K DArTag SNP panel (Zhao et al., 2023), developed specifically for alfalfa, a close relative of annual medics. A total of four samples (three single-plant and one bulked) for each accession were genotyped, with the exclusion of two reference accessions (PI 312480 – *M. monspeliaca* and W6 24404 – *M. minima*) to conform more readily to the 96-well genotyping platform format. It is worth noting that using this DArTag platform, for each marker locus, there could be varying numbers of microhaplotypes and SNP markers depending on the genetic diversity of the samples genotyped.

**Figure 5:**
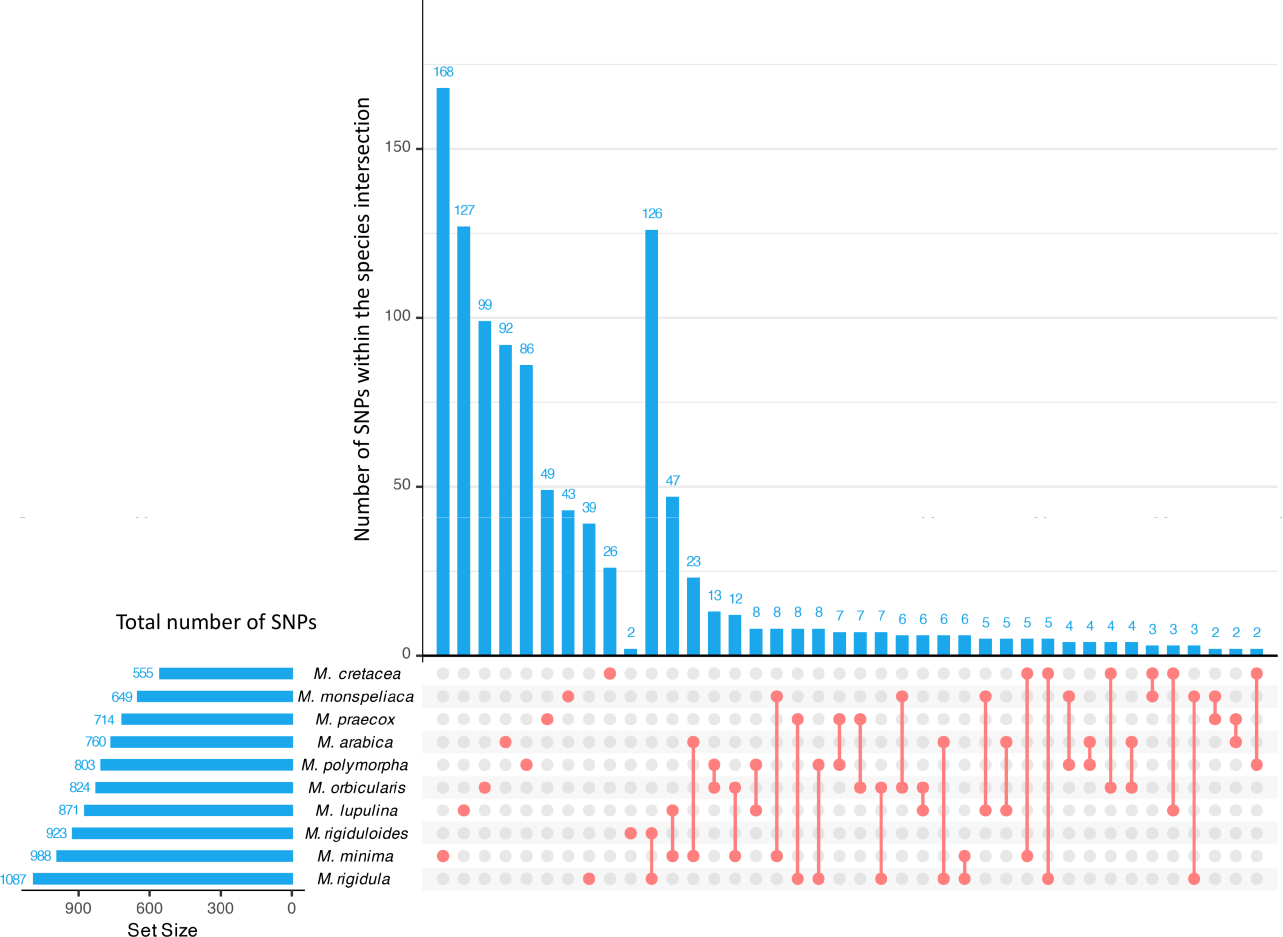
Upset plot of the intersection of polymorphic SNPs across different species. The horizontal bars represent total number of polymorphic SNPs found among the accessions within the corresponding species. The vertical bar plots represent the number of SNPs either unique to the specific species or common markers between two species indicated below the bars. The first ten vertical bars from the left to right represent SNPs unique to the species as indicated by the red dot whereas the remaining bars represent number of SNPs shared between two species indicated by red dot and the line segment.

An initial filtering for missing data was conducted at the microhaplotype level by requiring at least a total of 10 reads per microhaplotype to be retained for subsequent analyses. Because the 3K panel was generated from alfalfa, we expected missing data rates would be higher for these annual medics (diploids). In total, 2,396 marker loci from the 3K panel showed signals in at least 5% of the 752 samples genotyped. On average, each sample exhibited signals from approximately 1,475 marker loci (**Supplementary Table 4**). To discover a shared set of SNP markers present in all *Medicago* species studied, we performed pairwise comparisons among all 10 species (**Figure 5**). However, most of the comparisons had very few common SNP markers (<20), indicating a distinct species-specific genetic variation among the accessions studied. Therefore, we elected to identify marker loci present in >95% of the samples, for which we successfully discovered 448 marker loci that were evenly distributed across the genome (**Tables 1**, **2**). While the markers in these loci are shared among 95% of the samples, it is worth noting that some species-specific markers are valuable for the genetic characterization of different species (**Figure 5**). Hence, we utilized the larger marker dataset of 2,396 loci for further analysis.

**Table 1:** Maker loci count found across accessions based on the percentage of accessions in which they were present.

% of genotyped samples	Maker loci #
5	2396
10	2291
15	2178
20	2081
25	1988
30	1877
35	1792
40	1709
45	1609
50	1510
55	1415
60	1320
65	1228
70	1132
75	1010
80	890
85	748
90	622
**95**	**448**
100	61

A set of 2,396 loci can be found in 5% of all samples. A set of 448 loci can be found in 95% of the samples.

**Table 2:** Distribution of targeted loci across the eight chromosomes based on the 448-marker set found in 95% of samples in this study compared to the distribution of marker loci in the original 3K DArTag panel.

Chromosome	Loci # identified in 95% of samples	Loci # in 3K DArTag panel	Loci ratio identified in 95% of samples to 3K DArTag panel
chr1.1	53	414	0.128
chr2.1	54	364	0.148
chr3.1	52	419	0.124
chr4.1	67	426	0.157
chr5.1	64	390	0.164
chr6.1	31	210	0.148
chr7.1	56	367	0.153
chr8.1	71	410	0.173
**Total**	**448**	**3000**	**0.149**

### 3.3 Missing data rates

**Figure 6:**
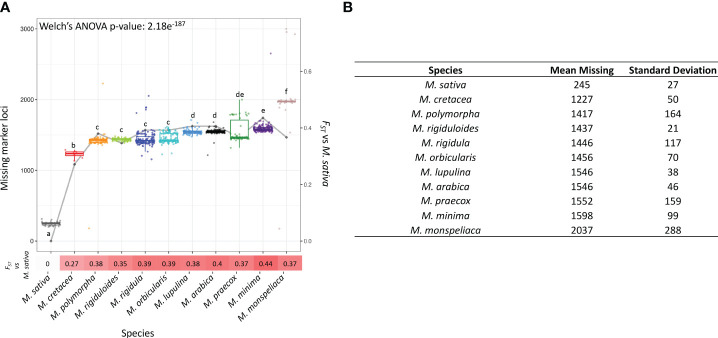
Summary of missing marker loci across different species. **(A)** Boxplot representing the distribution of the number of missing marker loci across 11 *Medicago* species. The letters in the boxplot indicate the grouping of species with significantly different missing rates evaluated by Games-Howell test (α < 0.05). The line and numbers with gradient color below the x-axis represents *F_ST_* values between *M. sativa* and the 10 diploid *Medicago* species. The x-axis represents different *Medicago* species. The primary y-axis denotes the number of missing marker loci whereas the secondary y-axis represents *F_ST_* value calculated between *M. sativa* and the 10 diploid *Medicago* species. **(B)** Statistics of missing rate of marker loci in each species.

When evaluating the number of missing marker loci across all the samples, an interesting pattern regarding the variation in the number of missing marker loci was found. We explored missing marker loci across all the species and identified significant differences (p-value: 1.13e^-47^) (**Figure 6**). *M. monspeliaca* exhibited the highest average count (2,037 ± 288) of missing marker loci, while *M. cretacea* displayed the lowest (1,227 ± 50), indicating genomic variations among species (**Figure 6B**). Furthermore, a general trend of increasing missing marker loci across the 10 species was observed with their increasing genetic distances (Fixation index, *F_ST_*) from *M. sativa* (**Figure 6A**).

### 3.4 Reproducibility and sensitivity of genotyping

**Figure 7:**
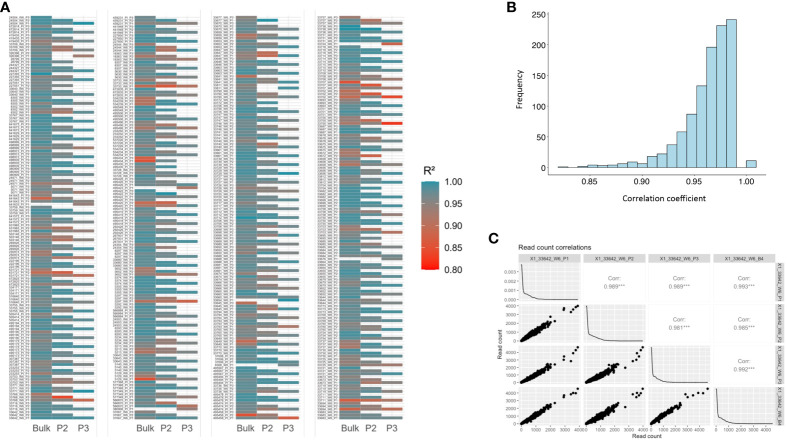
Correlations of read counts per marker loci among three single-plant samples and the bulked sample for each of the 192 accessions. **(A)** Heatmap representing correlations among single-plant samples (P1, P2, and P3) and the bulked sample for each of the 192 accessions. **(B)** Histogram of correlation coefficients among the single-plant samples and bulked samples. **(C)** Correlation matrix among three single-plant samples and the bulked sample of a selected *M. arabica* (W6 33642) accession.

The reproducibility and sensitivity (in terms of identifying rare alleles) of the genotyping results was also examined by calculating correlations among four samples (three single-plant samples and a bulked sample) for each accession. We observed a strong correlation among the four samples (average R^2^ = 0.965), indicating consistent genotyping results (**Figure 7** and **Supplementary Table 5**). Likewise, the three single-plant samples and the bulked sample demonstrated strong correlations (average R^2^ = 0.967) in read counts per marker locus, suggesting high reproducibility across the shared marker loci.

**Figure 8:**
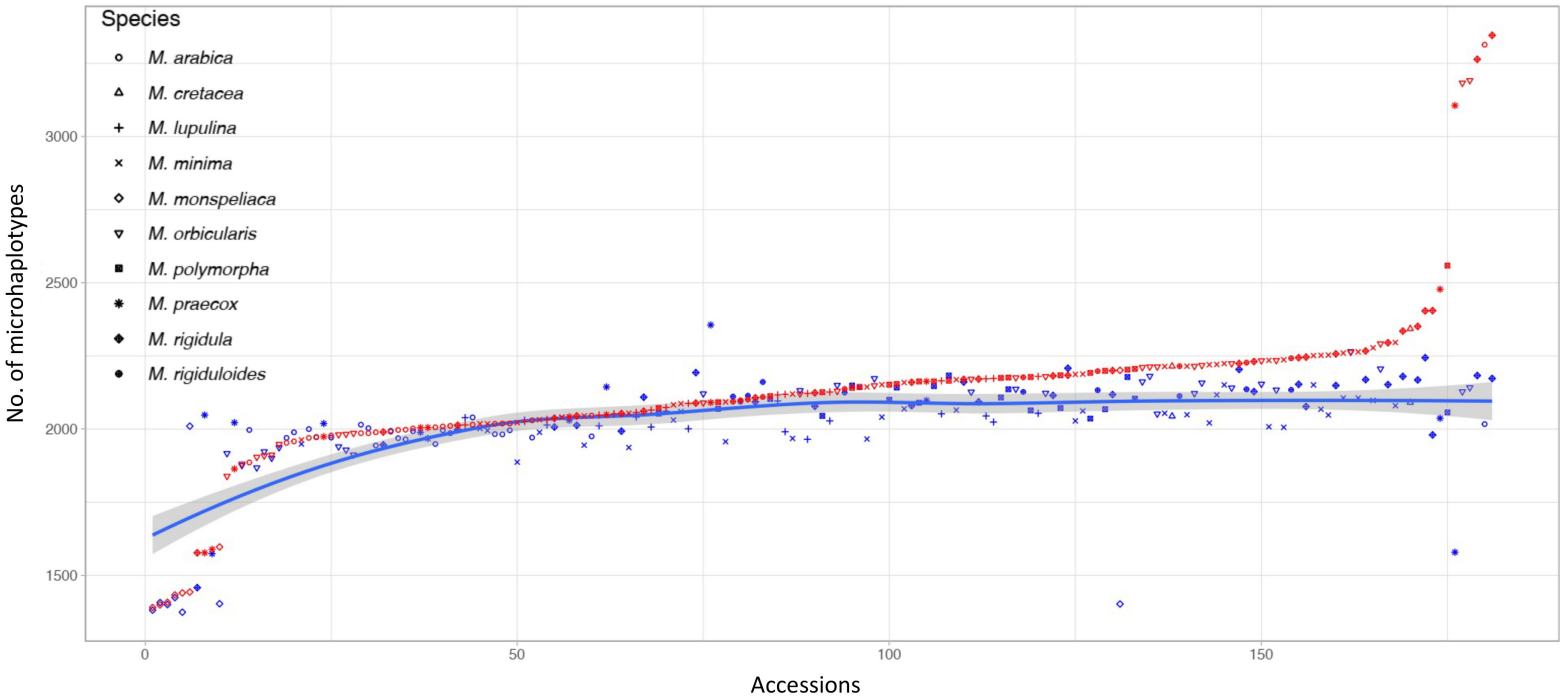
Scatter plot of sample-wise microhaplotype numbers in the single-plant (median of the three single-plant samples; blue dots) and bulked (red dots) samples for 181 accessions. The trend (blue curve) of microhaplotype numbers among single-plant samples was generated using a LOWESS regression.

To compare genetic diversity that can be captured by the 3K DArTag panel, the total number of microhaplotypes were estimated for single-plant and bulked samples for each accession (**Supplementary Table 6**). Overall, the single-plant and bulked samples had an average of 2,027 and 2,126 microhaplotypes, respectively, from all 2,396 marker loci (a total of 12,945 microhaplotypes). Of the evaluated 181 accessions (with three single-plant samples and one bulked sample), 147 of the bulked samples showed a greater number (1 to 1,527; 0.01% to 11.80%) of microhaplotypes than the corresponding single-plant samples. Only 34 single-plant samples showed higher numbers (1 to 567; 0.01% to 4.38%) of microhaplotypes than their corresponding bulked samples (**Figure 8**; **Supplementary Table 6**). A LOWESS regression was employed to perform microhaplotype number smoothing across the 181 accessions. This was done by using the median haplotype number from the three single-plant samples for each accession. This analysis revealed a prevailing trend of slightly fewer microhaplotypes in single-plant samples compared to their bulked counterparts (**Figure 8**). Nevertheless, upon consolidating microhaplotypes from all three single-plant samples for each accession, we observed an increased count of microhaplotypes in 171 accessions and an average of 190 (14.68%) more microhaplotypes identified in the consolidated single-plant samples across all accessions (**Supplementary Figure 1**; **Supplementary Table 6**).

To evaluate genetic diversity that can be captured at each marker locus, average proportion of microhaplotype numbers per marker locus were estimated across the entire panel and within each of the 10 species. Across the entire panel, 1,922 (80.22%) marker loci had more microhaplotypes identified in bulked samples than single-plant samples (**Supplementary Figure 2A**). Because significant differences in missing rate were observed across the 10 species, the same calculation was applied to each species. Overall, 28.83% to 73.77% of the marker loci had more microhaplotypes identified in bulked samples than single-plant samples (**Supplementary Figure 2B**). Across all 10 species, the proportion of marker loci that had elevated number of microhaplotypes in bulked samples was strongly correlated (*r* = 0.726) with the sample size of a species (**Supplementary Figure 3**).

### 3.5 Genotypic population structure

**Figure 9:**
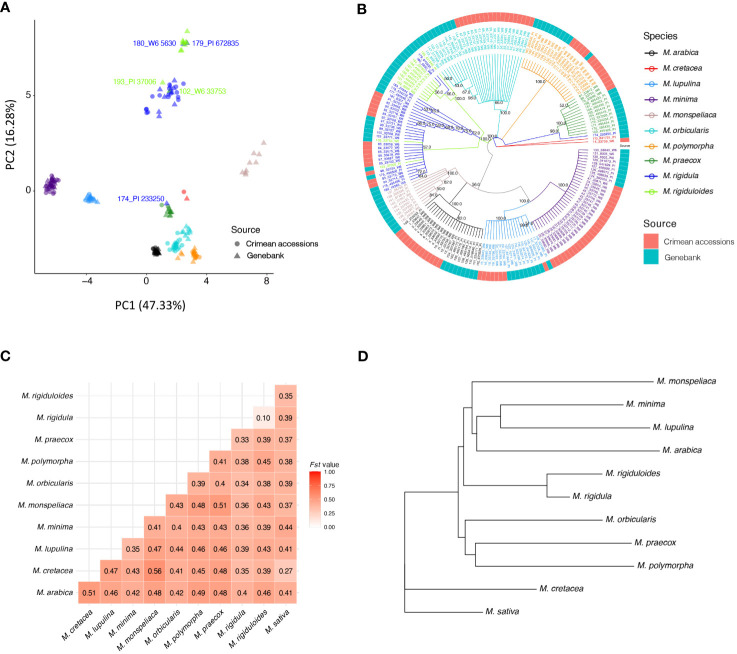
Population structure analysis of the accessions used in the study. **(A)** Principal component analysis using estimated allele dosages in 192 accessions. Possible mislabeled or misidentified accessions are listed in the figure. **(B)** Phylogenetic tree representing relationships among the 192 accessions. Different colors of accessions and branches represent unique species. The colors in the outer concentric circle represents the collection accession source. The numbers on the branches represent the bootstrap values (>50%). **(C)** Heatmap of pairwise *F_ST_* values among all 10 species used in the study and *M. sativa* accessions from Zhao et al. (2023). **(D)** Simplified species tree representing genetic relatedness of the *Medicago* species under study based on the neighbor joining tree constructed using pairwise *F_ST_* comparison with *M. sativa* as the root.

Population structure and relationship among the accessions underlying the observed genomic variations was investigated. To ensure a representative analysis, we selected a single plant replicate of each accession with the genome-wide highest average read depth (**Supplementary Table 7**) for further investigation. PCA was performed using dosage-based calls using both the entire set of marker loci and a subset of 448 marker loci that were evenly distributed across the genome and found in >95% of the genotyped accessions. PCA demonstrated distinct clustering patterns corresponding to different species (**Figure 9A**). Some accessions from different species exhibited close clustering, indicating higher genetic similarity among species, while others formed distinct clusters from the rest of the population, indicating greater genetic distinctiveness (**Figures 9A, C**). For instance, consistent with phenotypic PCA, *M. rigidula* accessions (30 accessions) and *M. rigiduloides* accessions (11 accessions) clustered nearby and had low *F_ST_* value (0.10). Conversely, *M. polymorpha* accessions clustered distantly from *M. monspeliaca* (10 accessions), which exhibited high *F_ST_* values (0.48) (**Figure 9A**). Generally, the accessions identified as the species clustered together regardless of the source of collection. Interestingly, we found five accessions grouped as outliers in unrelated species clusters. Two accessions labeled as *M. rigidula* (PI 672835 and W6 5630) were found to cluster with *M. rigiduloides*. Another labeled *M. rigidula* accession (PI 233250) was found to be closely associated with *M. praecox* accessions. Similarly, two *M. rigiduloides* accessions (W6 33753 and PI 37006) were found to cluster with *M. rigidula* accessions (**Figure 9A**). This indicates either a high similarity of the accession to other species, like an admixture, a possible mislabeling, or misidentification of the accession due to similar phenotypic characterization (**Figure 3**). Additionally, we successfully categorized most of the samples into distinct clusters representing various species by utilizing the 448 marker loci (**Supplementary Figure 4**). However, accessions belonging to species like *M. rigidula* and *M. rigiduloides* were not differentiated with the reduced set of markers, because these two species shared the most marker loci (126) and had very few unique markers (*M. rigidula*: 39 and *M. rigiduloides*: 2) (**Figure 5**). This indicates that the identified common set of marker loci can be applied only for general purposes, such as determining population structure in general and assigning distinct *Medicago* species.

The phylogenetic tree constructed using the 2,396 SNP markers yielded results consistent with the PCA. We found that the accessions grouped into clades corresponding to their species, with high bootstrap values (>50) (**Figure 9B**). Furthermore, we observed related species grouping closer in the phylogenetic tree consistent with the PCA and *F_ST_* values. For example, *M. minima* and *M. lupulina* were grouped relatively close in the PCA analyses and found to arise from the same branch in the phylogenetic tree. They also had low *F_ST_* values (0.35) (**Figure 9C**). Additionally, within the clade of the same species, *M. orbicularis*, accessions from the Crimean accessions formed separate branches, indicating genetic diversity within the clade (**Figure 9B** and **Supplementary Figure 5**). Notably, the *M. cretacea* accessions were not grouped with any other species, indicating their genetic distinctiveness from other species. It is worth noting that we only included two accessions of *M. cretacea*. Furthermore, consistent with PCA, we identified the same five accessions grouped in a different species clade than their original labels. Moreover, the phylogenetic tree constructed from pairwise *F_ST_* values (**Figure 9D**) also aligned with the other findings.

### 3.6 Crimea and Genebank accession comparison

To further evaluate the genetic diversity, we conducted a comparative analysis between accessions collected from Crimea and those reference accessions already in the NPGS. We observed a significant difference (p-value: 6.98e^-05^) in missing markers between the accessions collected in the Crimea and those collected from the NPGS collection (**Supplementary Figure 6**), indicating the presence of underlying genetic variation. Moreover, the PCA plot yielded a sub-clustering of accessions within species such as *M. orbicularis* based on their acquisition source (**Figure 9A** and **Supplementary Figure 5**). This grouping suggests the Crimea material added novel genetic diversity into the existing NPGS collection. Supporting this observation, we identified relatively high genetic dissimilarity for accessions collected for species like *M. orbicularis* (*F_ST_* = 0.130) and *M. praecox* (*F_ST_* = 0.113), which further underscores the impact of novelty in the collected germplasm (**Table 3** and **Supplementary Figure 5**).

**Table 3:** Estimated *F_ST_* between annual medic (*Medicago* spp.) accessions from Crimea and from the NPGS across different species.

Species	*F_ST_* (Gene bank vs Collection)
*M. arabica*	0.003
*M. cretacea*	–
*M. lupulina*	0.013
*M. minima*	0.009
*M. monspeliaca*	–
*M. orbicularis*	0.130
*M. polymorpha*	0.044
*M. praecox*	0.113
*M. rigidula*	0.021
*M. rigiduloides*	–

“-” denotes absence of analysis either due to lack of samples in either of the group or very few number of accessions.

## 4 Discussion

In this study, we successfully characterized genetic diversity and population structure in a subset of annual medic accessions collected and integrated into the NPGS collections using both phenotypic and molecular markers. Annual medics are important forage legumes used extensively in regions of domestication, where they have naturalized, and where they have been introduced as crops like in Australia (Crawford et al., 1989; Nichols et al., 2012). They are also related to alfalfa, and at least some have been used in breeding introgressing traits into this important crop (Irwin et al., 2010; Bingham, 2013; Humphries et al., 2021). Characterizing diversity in germplasm collections is an activity that helps inform curatorial management decisions while providing information to stakeholders. The genotyping research served as a proof of concept that DArTag markers developed in alfalfa could be used to genotype related annual medic species. The analyses conducted aided in the correct taxonomic identification and helped inform relationships between and among species when compared to a reference subset.

The findings of this study hold crucial significance for current agricultural practices and the production of annual medics. Through the comprehensive characterization of genetic diversity and population structure, we have provided valuable insights that can inform germplasm conservation strategies and breeding programs. The identification of unique genetic variants and their associations with specific phenotypic traits within the Crimean germplasm collection presents an opportunity to enhance the adaptability and productivity of annual medics and offer a foundation for targeted breeding programs. This knowledge is helpful for developing cultivars with improved traits, such as drought resistance, pest tolerance, and enhanced nutritional content, contributing to the sustainability and efficiency of forage legume production. The study also highlights the importance of preserving and exploring diverse germplasm collections to unlock hidden genetic potential, ensuring the continual improvement of annual medic crops.

Phenotypic traits describing phenology, plant, leaf/leaflet, flower, and fruit phenotypes were useful in showing differences among accessions within species, but clearly helped define the distinctions between species. Differences among accessions within species was greater in reference accessions when compared to those from Crimea and was likely due to their selection from a broad geographic area to be generally representative of each species. This was evident from PCA plots, too. In a few accessions, off-type plants were identified based on their phenotypes and eliminated from the average recorded descriptor trait. Also, in a few instances accessions appear to be mislabeled or misidentified with phenotypic descriptors matching other species being evaluated. In these situations where ‘errors’ were encountered, notes were taken and used for comparing to genotyping results. Also, when possible correct taxonomic assignment was assigned and amended in the NPGS Germplasm Resources Information Network (GRIN)-Global database.

In this research we utilized the recently published alfalfa 3K DArTag marker panel (Zhao et al., 2023) to genotype accessions to overcome the challenges often encountered when genotyping related species using marker panels developed for major crop species. Our findings demonstrated the applicability and effectiveness of the DArTag marker panel in genotyping the diverse annual medics, where targeted marker panels are unlikely to be available any time soon for these species. Despite the presence of a relatively high number of missing marker loci, the marker panel provided reliable genotyping results. The high correlation observed among single-plant samples and the bulked sample (**Figure 7**) for the same accession indicates the consistency and reproducibility of the marker panel, suggesting that replicates of each accession may not be necessary due to the high reproducibility. Furthermore, we noted a high correlation between the genotyping results of the bulked and single-plant samples, indicating that the bulked sample can also be used to obtain reliable and consistent genetic information from the panel. However, if rare microhaplotypes are critical for the study, it is highly recommended to genotype multiple single-plant samples instead of bulking them into one sample, as indicated by our findings where we observed an increased count of microhaplotypes in the consolidated single-plant samples than the bulk (**Supplementary Figure 1**; **Supplementary Table 6**). If similar read coverage could be achieved, it is possible that rare alleles could be detected in single plant samples, resulting in the observation of more microhaplotypes when combining read counts from all three single-plant samples in comparison with their respective bulked samples. We observed slightly elevated genetic diversity in the bulked samples of seven plants compared with a representative sample (median of the three) of three individual plants (**Figure 8**). With increased read depths (~two times higher), the 3K DArTag panel was able to capture elevated genetic diversity in the combined single-plant samples (**Supplementary Figure 1**). Thus, genotyping several single-plant samples separately could likely allow for the discovery of rare alleles, but at a higher cost and with some potential “genotyping noise” from paralogous amplification. In summary, with similar read depths, the 3K DArTag panel was able to capture the increased genetic diversity in bulked samples compared with single-plant samples, and genotyping several single-plant samples and studying them together could increase the possibility of identifying rare alleles, thus, discovering more existing genetic diversity for a species.

This marker panel could allow the efficient assessment of genetic diversity, population structure, and taxonomic confirmation in other medic species. Larger collections exist in the NPGS for *M. polymorpha* (747), *M. orbicularis* (383), *M. minima* (382), and *M. lupulina* (289) [Source: GRIN-Global https://npgsweb.ars-grin.gov/gringlobal) where these types of evaluations would be valuable. Taxonomic misidentification or mislabeling between closely related taxa like *M. rigidula* and *M. rigiduloides* could also be addressed. Marker panel utility would increase when reducing the taxonomic complexity of the species being evaluated to increase the number of successfully amplified/sequence loci (reducing missing loci) in common within a species or closely related species. These types of evaluations could be structured in such a way that closely related taxa could be included as well as outgroup species like alfalfa.

The presence of missing marker loci can be attributed to various factors, including variations in primer sites and the potential presence of structural variations in the targeted regions. Structural variations, such as insertions, deletions, or duplications, can significantly impact gene expression and protein functions, leading to variations in phenotype (Alonge et al., 2020; Sapkota et al., 2023). Therefore, the observed missing marker loci are possibly associated with the phenotypic natural variation observed in the species being evaluated, providing valuable insights into the underlying genomic variation contributing to the diverse phenotypes within the collection. Moreover, a noteworthy correlation was established between the frequency of missing marker loci and the degree of relatedness to *M. sativa*, for which the marker panel was originally developed. This correlation followed the anticipated pattern, with species that are evolutionarily closer to *M. sativa* exhibiting fewer missing marker loci, while those more distantly related displayed higher numbers (**Figures 6**, **9**). This observation underscores the predictive value of the marker panel’s efficiency based on a species’ proximity to the panel’s origin. Additionally, this also highlights the importance of considering species-specific genomic differences in marker development and genotyping analyses. In essence, the presence of missing marker loci not only sheds light on the potential genomic underpinnings of phenotypic diversity but also highlights the marker panel’s utility as a tool for efficiently assessing genetic relatedness among species, offering valuable guidance for its application in diverse genetic studies. Future investigations are necessary to evaluate potential biological or technical causes behind species-specific variations in missing data rates among different *Medicago* species, aiming for a comprehensive understanding of this phenomenon.

Although previous studies have employed various maker platforms for assessing germplasm genetic diversity (Brummer et al., 1991; Kidwell et al., 1994; Musial et al., 2002; Maureira et al., 2004; Djedid et al., 2021; Emami-Tabatabaei et al., 2021), mapping (Brummer et al., 1993; Brouwer and Osborn, 1999; Kaló et al., 2000; Choi et al., 2004; Badri et al., 2011; Gorton et al., 2012) and establishing trait associations in breeding programs (Barcaccia et al., 2000; Brouwer et al., 2000; Tavoletti et al., 2000; Badri et al., 2011; Gorton et al., 2012) of *Medicago* spp., these approaches have inherent limitations. Marker techniques such as RAPD, RFLP and AFLP are challenging to execute and often lack reproducibility. Microsatellite or SSR markers incur high development costs and generally exhibit low throughput, surveying only a limited number of loci (Putman and Carbone, 2014). GBS, while advantageous in generating many SNP markers, encounters challenges, especially in bioinformatic analyses, particularly for polyploid organisms like alfalfa or those lacking reference genomes (e.g., annual medics), leading to resource-intensive analysis processes (Bhatia et al., 2013; He et al., 2014; Rajendran et al., 2022; Reyes et al., 2022). The alfalfa DArT panel, however, offers a promising solution, addressing many of the limitations associated with these methodologies. Its amplicon-based targeted genotyping approach, as opposed to the more complex and resource-intensive techniques, allows for efficient SNP and microhaplotype identification. Notably, it offers a balance between high-throughput marker generation and the practical considerations of cost and ease of execution. The amplicon-based targeted genotyping approach of the DArT panel, as demonstrated in this study, proves advantageous for diverse *Medicago* species, offering an effective solution for genetic diversity assessment and breeding program applications. This makes the DArT platform a valuable addition to the molecular toolkit for the breeders.

In addition to the successful genotyping of annual medics using the alfalfa 3K marker panel, our study also revealed valuable insights into the phenotypic and genotypic diversity within the Crimean germplasm collection. The collection, assembled from the Crimean Peninsula of Ukraine, was specifically targeted to fill gaps in geographic coverage in the NPGS TFL collections. The diverse phenotypic traits observed in the collection highlight the richness of genetic resources that can be explored to use as crops, and for breeding and molecular biology applications. Our analysis revealed the presence of unique genetic diversity in accessions for certain species from the Crimean collection. These accessions exhibited distinct clustering patterns and genetic signatures, indicating their value in filling gaps in coverage in the existing collections, and as potential sources for breeding. The identification of such unique genetic variants within the collection also highlights the importance of preserving and studying diverse germplasm collections to unlock hidden genetic potential. Another important point to make was that a single accession of *M. cretacea*, a close alfalfa relative in the same taxonomic Section (*Medicago*), from Crimea and from the reference collections were included. That is because this is an underrepresented species in the collections that could be targeted for additional acquisitions.

Population structure analysis enables one to understand genetic diversity in each collection and identify the appropriate population for association mapping (Qiang et al., 2015). The population structure analysis conducted in our study played a crucial role in understanding the genetic diversity within species and identifying appropriate subpopulations for association mapping. The clustering of accessions based on both phenotype and genotype data generally agreed with the predicted evolutionary relationships among species. This consistency reinforces the reliability of the marker panel and its ability to capture the underlying genetic structure of the annual medics studied. Moreover, the clustering patterns also reflected the distinct genetic backgrounds of different species within the collection, providing important information for breeders and researchers interested in specific species or traits. Additionally, our study delved into the genetic relatedness and phylogeny among the various *Medicago* species and provided insights into the evolutionary relationships among the studied species. Notably, *M. cretacea* was found to be the closest, while *M. monspeliaca* was the furthest from *M. sativa*, a consistency observed in the respective number of missing marker loci within their accessions. Overall, both PCA and the phylogenetic tree analysis confirmed the species-level clustering and revealed additional insights into genetic relationships and diversity within and between species. While prior research has touched upon these aspects, the comprehensive inclusion of all these species in a single study is unique. Consequently, our study provides an overarching view of the genetic relatedness among the species in focus, addressing a pre-existing knowledge gap within the field.

The marker panel not only facilitated the characterization of genetic diversity but also helped in uncovering potentially misidentified, mislabeled or mixed-up samples within the collection. The similarity in phenotypes between many annual medic accessions and across different species can make it difficult to classify and assign taxonomy correctly. Conversely, a uniqueness in phenotypes of these few accessions allowed for individual accessions to be misclassified. By comparing the genotypic data with the known taxonomic identities, we were able to identify instances of misidentification or labeling errors, emphasizing the importance of reducing bias in sample management within germplasm collections. Accurate sample identification is crucial for the integrity of germplasm collections, and the marker panel provided an efficient tool for detecting discrepancies despite being phenotypically very similar. This marker panel stands out among several tools available for identifying potential mislabeling or mix-ups in samples. This resource and information are invaluable for guiding breeders or germplasm curators to conduct further validation and confirmation of the labeling of these accessions. Correcting these identification errors is essential for ensuring the reliability and usability of the collection for future research and breeding efforts.

Moving forward, our findings set the stage for future studies that can delve deeper into the genetic architecture of annual medics. The maker panel could also lead to the possible identification of specific genomic regions associated with desirable traits that may be beneficial in these annual medic crops or as possible sources of desirable introgression in alfalfa (Irwin et al., 2010; Bingham, 2013; Humphries et al., 2021). This insight calls for extensive genome-wide association studies (GWAS) connecting phenotypic features with genetic markers. Such studies could unravel the intricate relationships between genotype and phenotype, identifying key genomic regions associated with desirable traits. Notably, the genomic regions or candidate genes underlying such associated loci could serve as prime targets for gene-editing research, with the advantage of having naturally occurring alleles as proof of concept. Integrating genomic information with both traditional breeding approaches and modern biotechnological tools can expedite the development of improved cultivars with tailored traits, reducing the time and resources required for breeding cycles and improving efficiency. Moreover, considering the existence of other crop-specific DArT panels, opportunities exist to replicate our marker panel evaluation approach in wild crop relatives across other vital crops. This exploration could shed light on the performance of marker panels, such as DArTag, in related species, providing insights into their adaptability and reliability. Given the slim probability of dedicated platforms for crop wild relatives, utilizing versatile tools like DArTag becomes crucial. For many germplasm curators, this becomes an invaluable starting point for their genotyping initiatives. Furthermore, this evaluation strategy aids in developing approaches to incorporate wild crop-related species into cultivated breeding programs by illustrating the proximity of wild species to their cultivated counterparts. In essence, our study not only has implications for annual medics but also sets a precedent for broader applications in crop genetics and breeding.

Overall, our study is the first to demonstrate the successful application of phenotypic and genotypic characterization (using the alfalfa 3K marker panel) efforts in assessing the genetic diversity and population structure of annual medics. Our study not only advances our understanding of the genetic diversity and population structure of annual medics but also provides practical implications for crop breeding, and germplasm curation. The panel’s reliability, reproducibility, and ability to identify misidentified samples make it a valuable resource for future studies and breeding programs. The comprehensive understanding of phenotypic and genotypic diversity gained from this research lays the foundation for conservation strategies, possible targeted breeding efforts, and functional genomics studies in annual medics. By harnessing the genetic resources present in the Crimean collection and leveraging the power of marker-assisted selection, researchers and breeders can accelerate the development of improved cultivars with enhanced traits, leading to advancements in forage legume agriculture.

## Data availability statement

The data presented in the study are deposited in the figshare data repository, accessed through the link https://figshare.com/articles/dataset/Genotype_and_Phenotype_data/25458262. All phenotypic descriptor data and genotype data are provided in the **Supplementary Tables**. Phenotype data are available in **Supplementary Table S3** and genotype data are available as **Supplementary Table S8**. All phenotypic descriptor data are also uploaded to the GRIN-Global database and associated with accessions being evaluated.

## Author contributions

DZ: Writing – original draft, Writing – review & editing, Data curation, Formal Analysis, Investigation, Methodology, Software, Validation, Visualization. MSa: Writing – original draft, Writing – review & editing, Data curation, Formal Analysis, Investigation, Methodology, Software, Validation, Visualization. ML: Writing – review & editing, Formal Analysis, Investigation, Software, Visualization. CB: Writing – review & editing, Conceptualization, Project administration, Supervision. MSh: Writing – review & editing, Conceptualization, Funding acquisition, Project administration, Resources, Supervision. SG: Writing – review & editing, Conceptualization, Resources. BI: Conceptualization, Data curation, Formal Analysis, Funding acquisition, Investigation, Methodology, Project administration, Resources, Supervision, Visualization, Writing – original draft, Writing – review & editing.
