# Supplementary material for: Genetic diversity, population structure, and taxonomic confirmation in annual medic (Medicago spp.) collections from Crimea, Ukraine
Source: Front Plant Sci. 2024 Apr 3;15:1339298. doi: 10.3389/fpls.2024.1339298 (PMC11021755; doi:10.3389/fpls.2024.1339298)
Supplement: Supplementary file 1 [file DataSheet_1.zip › Figures S1 - S7.DOCX]

Supplementary Material

**Genetic diversity, population structure, and taxonomic confirmation in annual medic (*Medicago* *spp*.) collections from Crimea, Ukraine**

Dongyan Zhao^1†^, Manoj Sapkota^1†^, Meng Lin^1^, Craig Beil^1^, Moira Sheehan^1^, Stephanie Greene^2^, Brian M. Irish^3*^

*** Correspondence:** Brian M. Irish: [brian.irish@usda.gov](mailto:brian.irish@usda.gov)

# Supplementary Figures and Tables

## Supplementary Figures

**Supplementary** **Figure 1:** Scatter plot of sample-wise microhaplotype numbers in combined single-plant (pooling sequences from all three single plants; blue dots) and bulked (red dots) samples for 181 accessions. The trend of microhaplotype numbers among combined single-plant samples was generated using LOWESS regression.

**Supplementary** **Figure 2:** Scatter plot of the proportion of microhaplotypes identified per marker among single-plant samples and bulked samples (A) across the entire panel and (B) within each of the 12 species. Red dots represent markers with elevated proportion of microhaplotypes identified in bulked samples and green dots vice versa.

**Supplementary** **Figure 3:** Scatter plot of the proportion of markers showing elevated number of microhaplotypes in bulked samples in a species and the sample size of the investigated species. The Pearson’s correlation coefficient is estimated in R.

**Supplementary** **Figure 4:** PCA using only 448 marker loci.


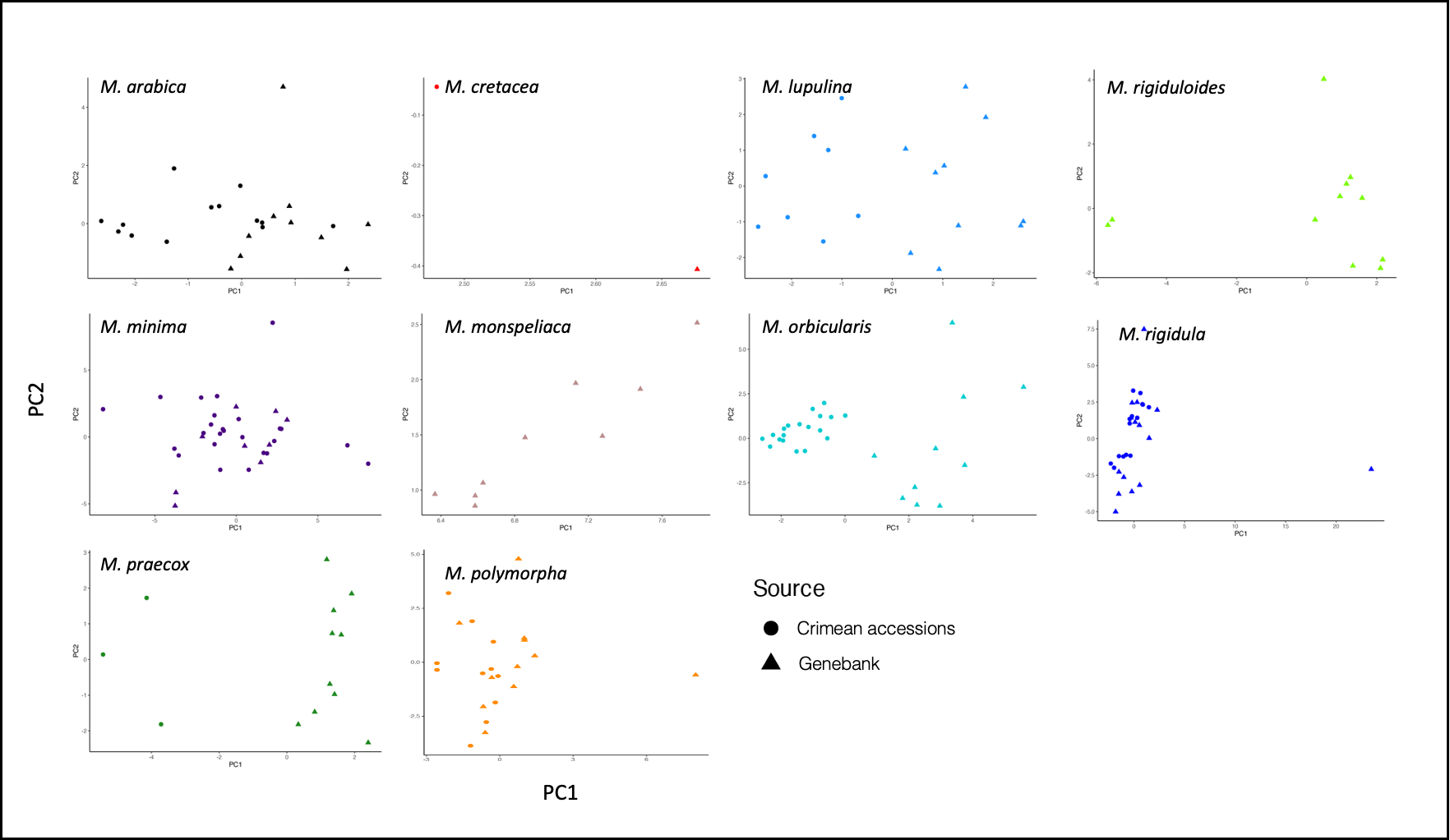


**Supplementary** **Figure 5:** Principal component analysis of accessions under each of the 10 species. Different shapes represent the source of collection.


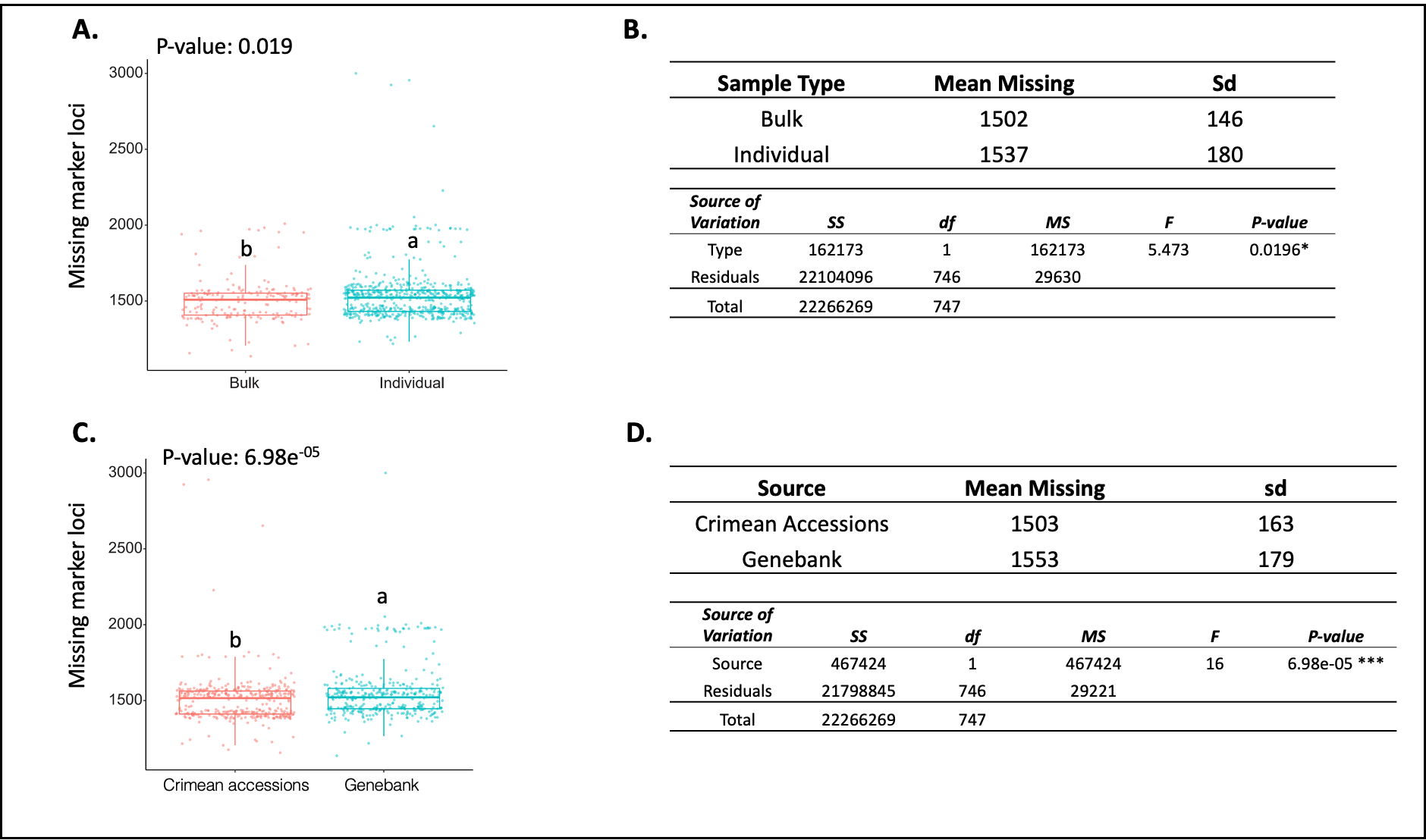


**Supplementary** **Figure 6:** Distribution of missing marker loci and summary statistics of the distribution between single-plant and bulked samples (A and B) and between accessions from Crimea and Genebank collection (C and D).

**Supplementary** **Figure 7:** Phylogeny tree representing relationship accessions from Crimea and *Medicago* accessions. Different colors of accessions and branches represent different species. The outer concentric circle represents the source of collection of accessions. The numbers on the branches represent the bootstrap values.

## Supplementary Tables

**Supplementary Table 1:** List of accessions used in the study with all their passport information.

**Supplementary** **Table 2:** List of phenotypic descriptors

**Supplementary** **Table 3:** Phenotypes observed for all the accession under study.

**Supplementary** **Table 4:** Summary of missing marker loci of all the accessions

**Supplementary** **Table 5:** Correlation across the replicates across all samples

**Supplementary** **Table 6:** Total number of microhaplotypes in a representative single-plant sample (median of three single-plant samples), combined single-plant sample (by pooling sequences from three single-plant samples) and the bulked sample in each of the 181 accessions.

**Supplementary** **Table 7:** Summary table of average read depth across all samples.

**Supplementary Table 8:** MADC report of all the genotyped accession
